# Supplementary material for: Collectively stabilizing and orienting posterior migratory forces disperses cell clusters in vivo
Source: Nat Commun. 2020 Sep 8;11:4477. doi: 10.1038/s41467-020-18185-2 (PMC7479147; doi:10.1038/s41467-020-18185-2)
Supplement: Supplementary file 1 — Supplementary Information [file 41467_2020_18185_MOESM1_ESM.pdf]

Supplementary Information

Collectively stabilizing and orienting posterior migratory forces disperses cell clusters *in vivo*

Lin et al.

Fig. S1- Tail phenotypes during PGC dispersal

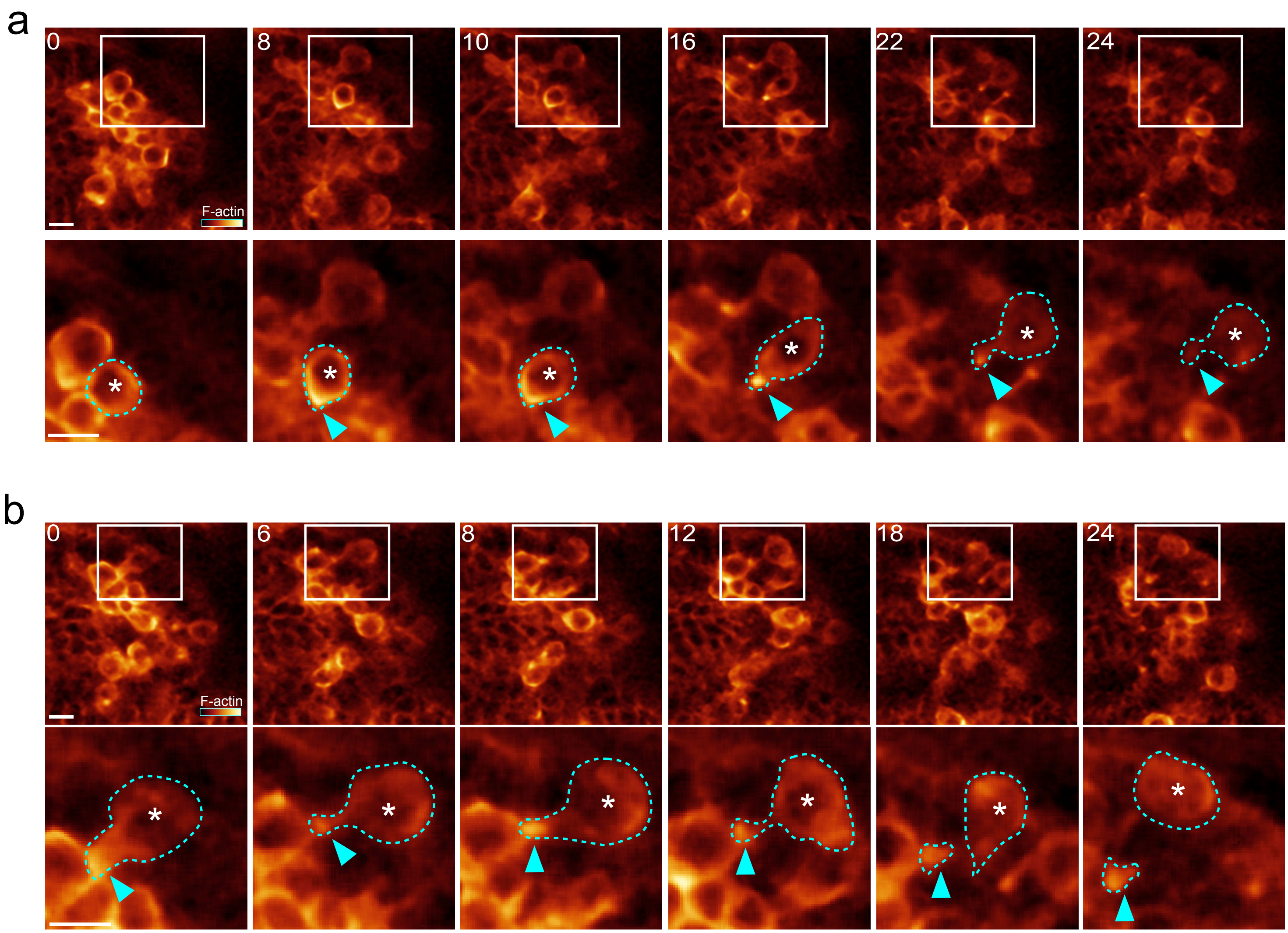

a-b, Two photon timelapse imaging of different Z slices from the WT PGC cluster shown in Figure 1 during dispersal, highlighting F-actin foci at PGC tails which are retained (**a**) or detached (**b**). F-actin is visualized with lifeact-tdTomato, which is pseudocolored with the indicated color bar in the first image (Intensity ranges- 662 - 13000 (**a**), 680 to 11000 (**b**)). The white rectange indicates the subregion expanded in the bottom row. Cyan dashed lines outline PGCs, white asterisks track PGCs, and cyan arrows mark PGC tails. Representative images are from n = 6 embryos. Times indicated are in minutes. Scale bars, 10 μm.

Fig. S2- Characterization of the Anillin RhoA-GTP binding domain

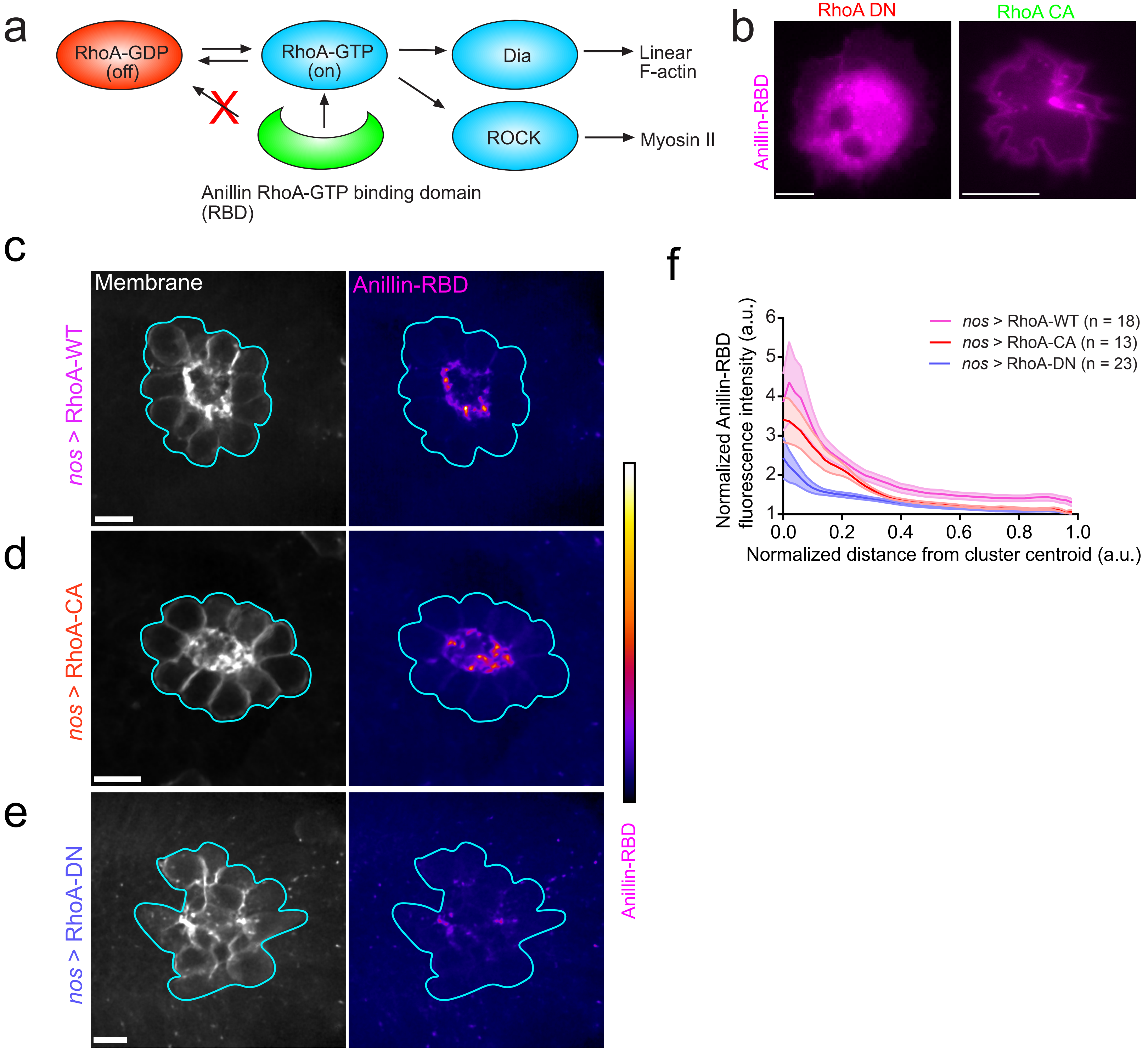

a, Schematic depicting simplified RhoA pathway and how the RhoA-GTP binding domain (RBD) of Anillin selectively interacts with GTP bound (active) RhoA. **b**, Anillin-RBD-tdTomato localization in S2 cells transfected with RhoA G14V (Constitutively active (CA)) and RhoA T19N (Dominant negative (DN)). The Anillin-RBD is cytoplasmic when RhoA DN is co-expressed, while it is enriched along the membrane when RhoA CA is co-expressed. Representative image is from n = 2 independent transfections. **c-e**, Two photon imaging of individual Z slices from the center of Anillin-RBD expressing PGC clusters which overexpress (**c**) RhoA-WT, (**d**) RhoA-Constitutively Active (CA), or (**e**) RhoA-Dominant Negative (DN). A membrane marker is also expresssed (tdKatushka2-CAAX). The Anillin-RBD is pseudocolored with the color bar on the right (Intensity range- 73 to 1000 in **c-e**). Representative images are from n = 18 embryos in **c**, n = 13 embryos in **d**, and n = 23 embryos in **e**. **f**, Quantification of normalized Anillin-RBD intensity relative to distance from the cluster centroid from PGC clusters overexpressing the indicated RhoA constructs. Number of clusters are indicated. Shaded regions represent SEM. All scale bars, 10 μm.

Fig. S3- Rac1 and Cdc42 binding domains are not polarized in PGC clusters

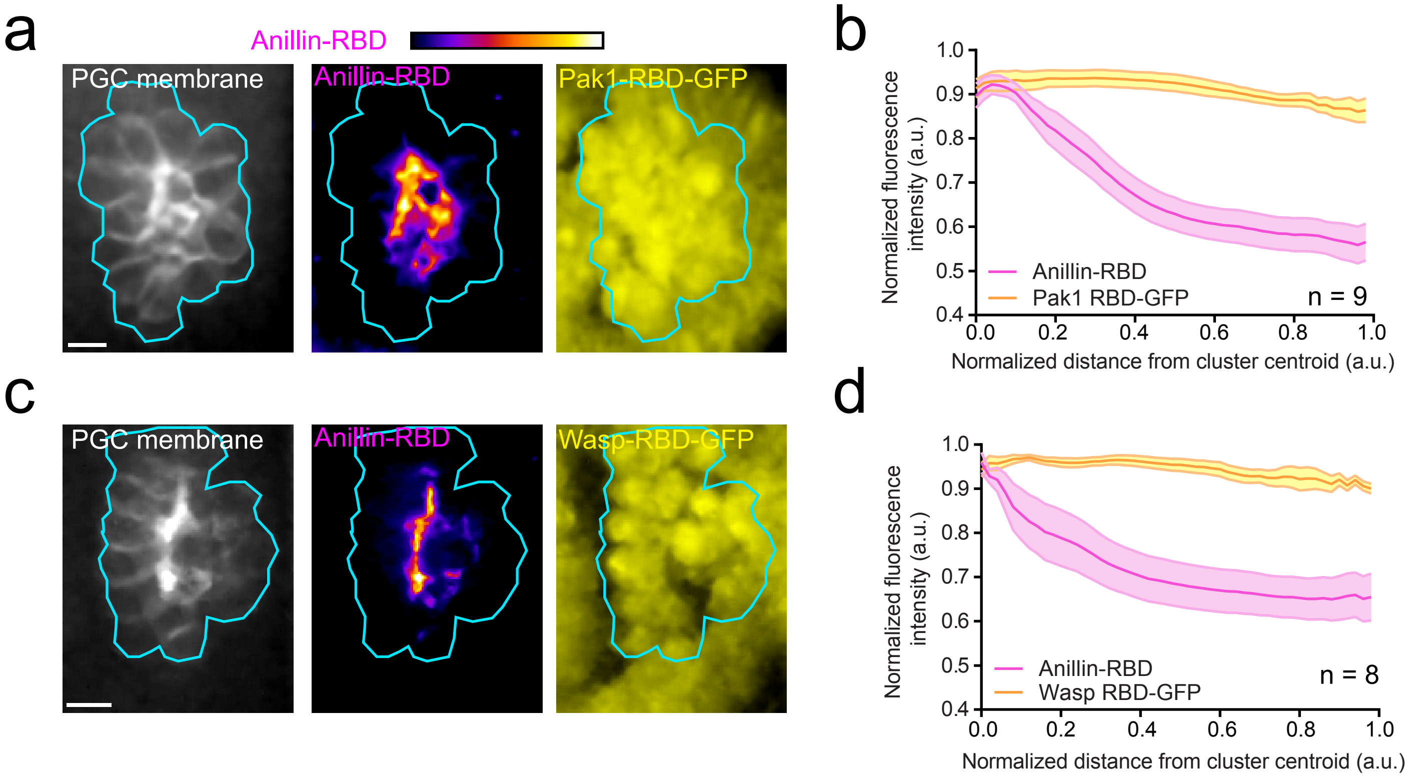

a, Two photon imaging of a representative WT PGC cluster expressing the Anillin-RBD, Pak1-Rho binding domain (RBD)-GFP, and membrane marker (tdKatushka2-CAAX). A single Z slice is shown. Anilllin-RBD is pseudocolored with color bar above (Intensity range- 1689 - 3771). Representative images are from n = 9 embryos. **b**, Quantification of normalized fluorescence intensity of the Anillin-RBD and Pak1-RBD-GFP in PGC clusters relative to the distance from the cluster centroid. Number of embryos are indicated. Shaded regions are SEM. **c**, Two photon imaging of a representative WT PGC cluster expressing the Anillin-RBD, Wasp-RBD-GFP, and membrane marker (tdKatushka2-CAAX). A single Z slice is shown. Anilllin-RBD is pseudocolored with color bar above **a** (Intensity range- 1108 - 3508). Representative images are from n = 8 embryos. **d**, Quantification of normalized fluorescence intensity of the Anillin-RBD and Wasp-RBD-GFP in PGC clusters relative to the distance from the cluster centroid. Number of embryos are indicated. Shaded regions are SEM. All scale bars, 10 μm.

Figure S4. Myosin II dynamics in WT and *tre1* PGC clusters

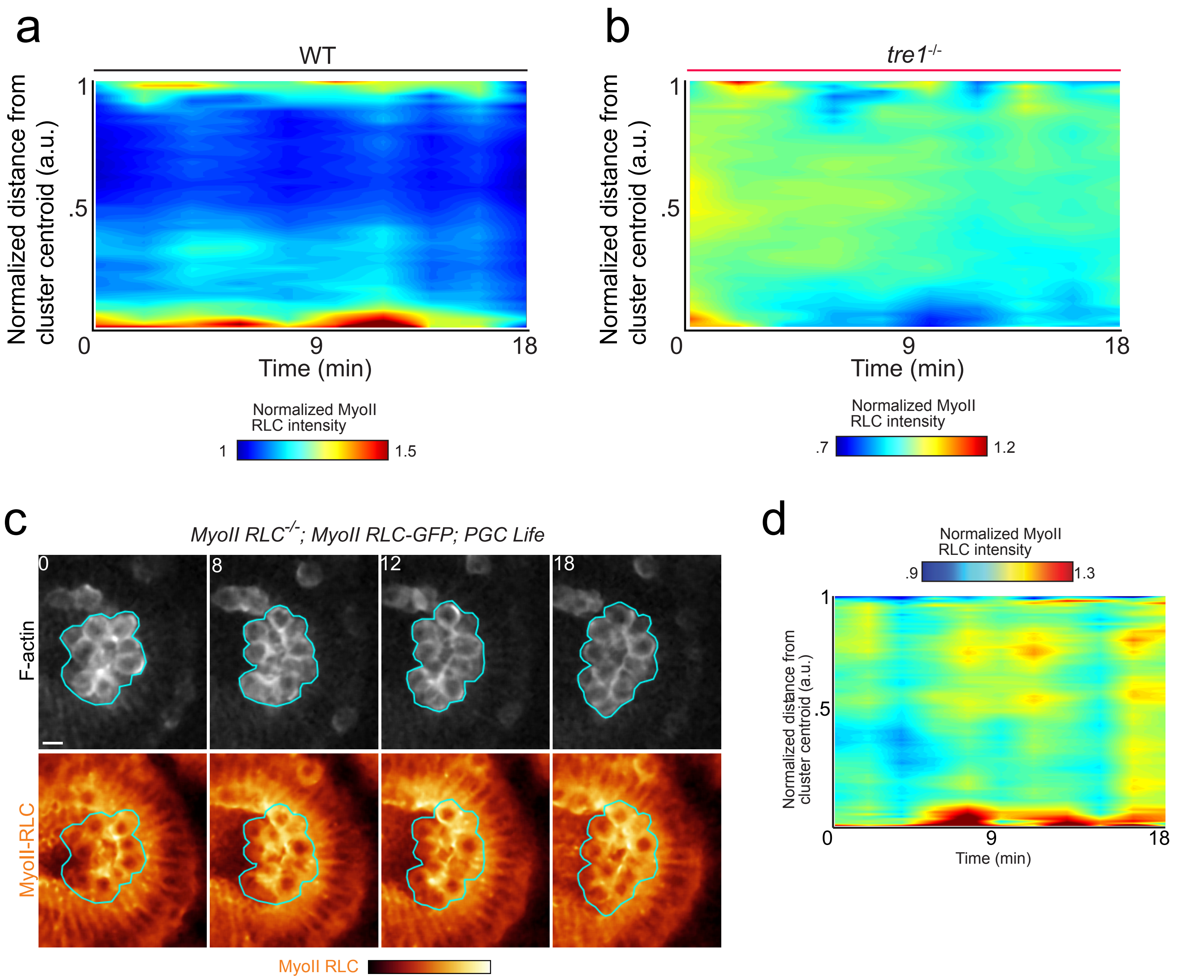

a-b, Quantification of normalized Myosin II RLC-GFP intensity vs. distance from the cluster centroid over time. These are the individual myosin II heatmaps from the WT (**a**) or $tre1^{-/-}$ (**b**) PGC clusters shown in Fig. 3e (**a**) and Fig. 3f (**b**). Myosin II RLC-GFP is pseudocolored with the indicated color bar on bottom. Note that the scaling is different between **a** and **b** to visualize dynamics. The enrichment near the cluster center in **a** is not present in **b**. **c,** Two photon timelapse imaging of a representative WT PGC cluster for 18 minutes prior to endoderm EMT with the indicated genotype on top. All myoII RLC provided to the organism is GFP tagged. A single Z slice is shown over time. Myosin II RLC-GFP is pseudocolored with color bar on bottom (Intensity range- 4000 - 11000). Representative images are from n = 5 embryos. Times are in minutes. Scale bars, 10 μm. **d**, Quantification of normalized Myosin II RLC-GFP intensity vs. distance from the cluster centroid over time from the cluster in **c**. The heatmap is interpolated. Myosin II RLC-GFP is pseudocolored with the indicated color bar on top.

Fig. S5- Optimization of 3'UTR for conditional overexpression in PGCs

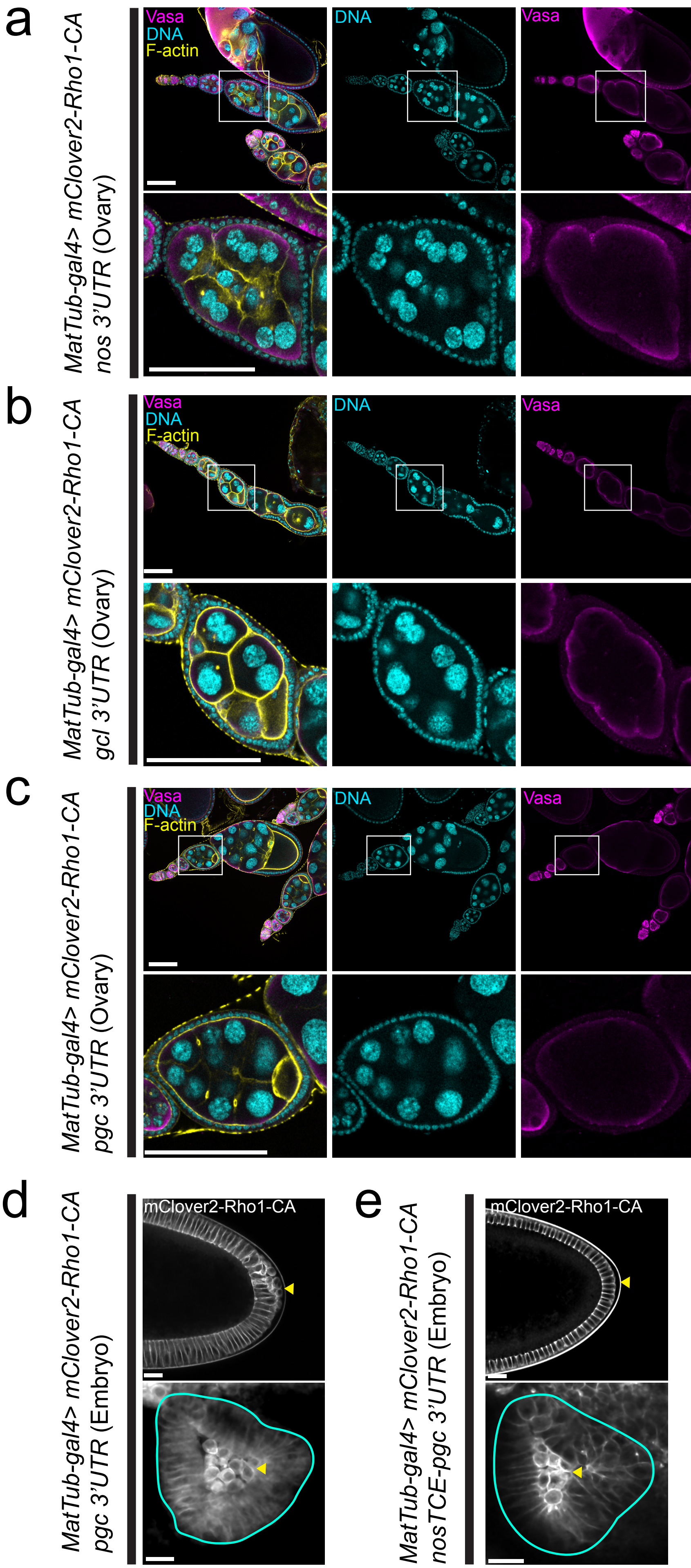

a-c, Representative ovariole from females of the indicated genotypes. 5 females were dissected from each genotype. Ovaries from females overexpressing mClover2-Rho1-constitutively active (CA) with the *nos* 3'UTR (**a**) or *gcl* 3'UTR (**b**) had multinucleated nurse cells with aberrant morphology. These females were sterile. Ovaries from females overexpressing mClover2-Rho1-CA with the *pgc* 3'UTR were normal and could produce eggs. Scale bars, 100 μm. **d-e**, Representative stage 5 (top) and stage 7 (bottom) embryos laid by females of the indicated genotypes. Posterior is to the right. 11 stage 5 embryos and 7 stage 7 embryos were imaged for (**d**), while 11 stage 5 embryos and 8 stage 7 embryos were imaged for (**e**). Yellow arrows in top embryos indicate the posterior where PGCs are present in (**d**) and absent in (**e**). 4/11 stage 5 embryos had no PGCs in (**e**). Yellow arrows in bottom embryos indicate PGCs. Cyan highlights the endoderm. Note that the transgene is more enriched in PGCs relative to the endoderm in (**e**). Scale bars, 20 μm.

Fig. S6- E-cadherin remains present on PGC membranes before and after endoderm EMT

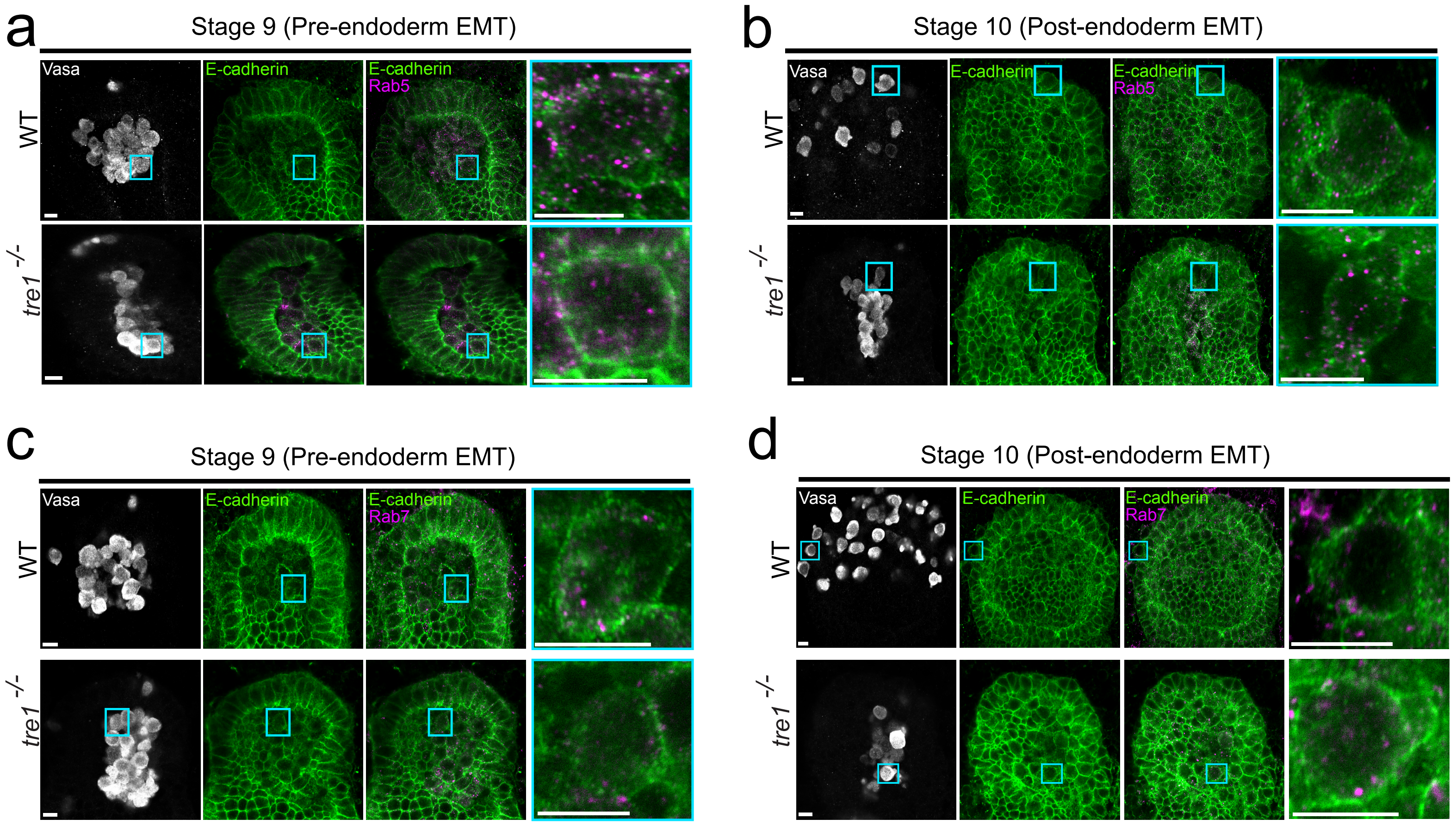

a-b, Confocal imaging of a single Z slice from representative WT and *tre1*$^{-/-}$ embryos stained prior to endoderm EMT (**a**, stage 9) and after endoderm EMT (**b**, stage 10) for Vasa, Ecadherin, and Rab5. Representative images are from n= 7 WT and n = 4 *tre1*$^{-/-}$ embryos in **a** and n = 4 WT and n = 6 *tre1*$^{-/-}$ embryos in **b**. **c-d**, Confocal imaging of a single Z slice from representative WT and *tre1*$^{-/-}$ embryos stained prior to endoderm EMT (**c**, stage 9) and after endoderm EMT (**d**, stage 10) for Vasa, Ecadherin, and Rab7. Representative images are from n= 7 WT and n = 8 *tre1*$^{-/-}$ embryos in **c** and n = 9 WT and n = 5 *tre1*$^{-/-}$ embryos in **d.** Scale bars, 10 μm.

	**Supplementary Table 1**	
**Figure**	**Genotype**	**Source**
1A, C	nos-Lifeact-tdTomato-P2A-tdKatushkta2-CAAX	**This study**
1B	tre1$^{ep\Delta5}$; nos-Lifeact-tdTomato-P2A-tdKatushkta2-CAAX	
1D, F-H	Matα-gal4::vp16 (67)/UASp(FRT.His2B-GFP)His2B-mKO;<br>nos-Lifeact-tdTomato-p2a-tdKatushkta2-CAAX	Matα-gal4::vp16 (67)- **BL7062**<br>UASp(FRT.His2B-GFP)His2B-mKO- **Xin Chen**[1]
1E, F-H	tre1$^{ep\Delta5}$; Matα-gal4::vp16 (67)/UASp(FRT.His2B-GFP)His2B-mKO; nos-Lifeact-tdTomato-p2a-tdKatushkta2-CAAX	
2A,C	nos-tdTomato-Anillin-RBD-P2A-tdKatushka2-CAAX/+	nos-tdTomato-Anillin-RBD-P2A-tdKatushka2-CAAX[2]
2B,C	tre1$^{ep\Delta5}$; nos-tdTomato-Anillin-RBD-P2A-tdKatushka2-CAAX/+	
2D,E	Matα-gal4::vp16 (67)/P{UASp-dia.RBD-GFP}; nos-tdTomato-Anillin-RBD-P2A-tdKatushka2-CAAX/+	P{UASp-dia.RBD-GFP}- **BL52291**
2F,G,I	nos-tdTomato-Anillin-RBD-P2A-tdKatushka2-CAAX/P{sqh-GFP-Rok}30	P{sqh-GFP-Rok}30- **BL52289**
2H,I	tre1$^{ep\Delta5}$; nos-tdTomato-Anillin-RBD-P2A-tdKatushka2-CAAX/P{sqh-GFP-Rok}30	
3A, C-D	nos-tdTomato-Anillin-RBD-P2A-tdKatushka2-CAAX/+;<br>P{sqh-GFP.RLC}3/+	P{squ-GFP.RLC}3- **BL57145**
3B, C-D	tre1$^{ep\Delta5}$; nos-tdTomato-Anillin-RBD-P2A-tdKatushka2-CAAX/+; P{sqh-GFP.RLC}3/+	
3E, H	nos-Lifeact-tdTomato-P2A-tdKatushka2-CAAX;<br>P{sqh-GFP.RLC}3	
3F, H	tre1$^{ep\Delta5}$; nos-Lifeact-tdTomato-P2A-tdKatushka2-CAAX; P{sqh-GFP.RLC}3	
3I-J	nos-Lifeact-tdTomato-P2A-tdKatushka2-CAAX; MyoII::3XGFP	MyoII::3xGFP- **Yohanns Bellaïche**[3]
4B, D-I	**Donor**- nos-Lifeact-tdTomato-P2A-tdKatushka2-CAAX; MyoII::3XGFP<br>**Host**- Tud^{A36-38}/Tud^{1}; nos-Lifeact-tdTomato-P2A-tdKatushka2-CAAX/+	Tud^{A36-38}, Tud^{1}- **Ruth Lehmann**
4C, D-I	**Donor**- tre1$^{ep\Delta5}$; nos-Lifeact-tdTomato-P2A-tdKatushka2-CAAX; MyoII::3XGFP<br>**Host**- Tud^{A36-38}/Tud^{1}; nos-Lifeact-tdTomato-P2A-tdKatushka2-CAAX/+	
5A,C-E	y^{1} w* cv^{1} sqh^{AX3}; P{w[+mC]=sqh-GFP.RLC}2;<br>UASp-degradFP- *nos* TCE-*pgc* -3'UTR, nos-Lifeact-tdTomato-P2A-tdKatushka2-CAAX/+ (female)<br>X<br>P{nullo-GAL4.G}5.20, P{w[+mC]=UAS-sqh.WT}TM1 (male)	y1 w* cv1 sqhAX3; P{w[+mC]=sqh-GFP.RLC}2- **BL57144**<br>UASp-degradFP-*nos* TCE-*pgc* 3'UTR- **This study**<br>P{nullo-GAL4.G}5.20- **BL26875**<br>P{w[+mC]=UAS-sqh.WT}TM1- **DGRC 109808**
5B,C-E	y^{1} w* cv^{1} sqh^{AX3}; P{w[+mC]=sqh-GFP.RLC}2;<br>UASp-degradFP-*nos* TCE-*pgc* 3'UTR, nos-Lifeact-tdTomato-P2A-tdKatushka2-CAAX/<br>P{w[+mC]=matalpha4-GAL-VP16}V37, P{sqh-GFP.RLC}3 (female)<br>X<br>P{nullo-GAL4.G}5.20, P{w[+mC]=UAS-sqh.WT}TM1 (male)	P{w[+mC]=matalpha4-GAL-VP16}V37- **BL7063**
5F-G	P{GAL4::VP16-nos.UTR}CG6325^{MVD1} (female) (Experiment)<br>X<br>w[*]; P[w+, UASp-CIBN::pmGFP]; P[w+, UASp-RhoGEF2-CRY2::mCherry] (male)	P{GAL4::VP16-nos.UTR}CG6325^{MVD1}- **BL4937**<br>P[w+, UASp-CIBN::pmGFP], P[w+, UASp-RhoGEF2-CRY2::mCherry] - **Stefano De Renzis**[4]
5F-G	P{GAL4::VP16-nos.UTR}CG6325^{MVD1} (female) (Control)<br>X<br>w[*]; P[w+, UASp-CIBN::pmGFP] (male)	
6A, C	**Donor**- Ecad::3xGFP; nos-Lifeact-tdTomato-P2A-tdKatushka2-CAAX<br>**Host**- nos-Lifeact-tdTomato-P2A-tdKatushka2-CAAX	Ecad::3xGFP- **Yohanns Bellaïche**[3]
6B, C	**Donor**- tre1$^{ep\Delta5}$; Ecad::3xGFP; nos-Lifeact-tdTomato-P2A-tdKatushka2-CAAX<br>**Host**- tre1$^{ep\Delta5}$; nos-Lifeact-tdTomato-P2A-tdKatushka2-CAAX	
6D, F	**Control**- Matα-gal4::vp16 (67)/+; nos-Lifeact-tdTomato-P2A-tdKatushka2-CAAX/+<br>**Ecadherin-OE**- Matα-gal4::vp16 (67)/UASp-DE-cadherin-mClover2-*nos* TCE-*pgc* 3'UTR; nos-Lifeact-tdTomato-P2A-tdKatushka2-CAAX/+	UASp-DE-cadherin-mClover2-*nos* TCE-*pgc* 3'UTR- **This study**
6E, G	**Control**- MatTub-gal4/+; nos-Lifeact-tdTomato-P2A-tdKatushka2-CAAX/+<br>**Neuroglian-OE**- MatTub-gal4/UASp-Neuroglian-mClover2-*nos* TCE-*pgc* 3'UTR; nos-Lifeact-tdTomato-P2A-tdKatushka2-CAAX/+	MatTub-gal4- **This study**<br>UASp-Neuroglian-mClover2-*nos* TCE-*pgc* 3'UTR- **This study**
6H, J	**Control**- tre1$^{ep\Delta5}$; Matα-gal4::vp16 (67)/+; nos-Lifeact-tdTomato-P2A-tdKatushka2-CAAX/+<br>**Ecadherin-OE**- tre1$^{ep\Delta5}$; Matα-gal4::vp16 (67)/UASp-DE-cadherin-mClover2-*nos* TCE-*pgc* 3'UTR; nos-Lifeact-tdTomato-P2A-tdKatushka2-CAAX/+	
6I, J	**Control**- tre1$^{ep\Delta5}$; MatTub-gal4/+; nos-Lifeact-tdTomato-P2A-tdKatushka2-CAAX/+<br>**Neuroglian-OE**- tre1$^{ep\Delta5}$; MatTub-gal4/UASp-Neuroglian-mClover2-*nos* TCE-*pgc* 3'UTR; nos-Lifeact-tdTomato-P2A-tdKatushka2-CAAX/+	
7A,C, E-G	Donor- nos-Lifeact-tdTomato-P2A-tdKatushka2-CAAX<br>Host- tre1$^{ep\Delta5}$, nos-EGFP::moe; nos-EGFP::moe	nos-EGFP::moe- **Ruth Lehmann**

7B,D, E-G	Donor- nos-Lifeact-tdTomato-P2A-tdKatushka2-CAAX<br>Host- nos-EGFP::moe	
Fig. S1A,B	nos-Lifeact-tdTomato-P2A-tdKatushkta2-CAAX	
Fig. S2C,F	nos-tdTomato-Anillin-RBD-P2A-tdKatushka2-CAAX/+; P{GAL4::VP16-nos.UTR}CG6325MVD1 (Female)<br>X<br>w[*]; P{w[+mC]=UAS-Rho1.W}3	P{w[+mC]=UAS-Rho1.W}3- **BL28872**
Fig. S2D,F	nos-tdTomato-Anillin-RBD-P2A-tdKatushka2-CAAX/+; P{GAL4::VP16-nos.UTR}CG6325MVD1 (Female)<br>X<br>w[*]; P{w[+mC]=UAS-Rho1.V14}2.1 (male)	P{w[+mC]=UAS-Rho1.V14}2.1- **BL8144**
Fig. S2E,F	nos-tdTomato-Anillin-RBD-P2A-tdKatushka2-CAAX/+; P{GAL4::VP16-nos.UTR}CG6325MVD1 (Female)<br>X<br>w[*]; P{w[+mC]=UAS-Rho1.N19}2.1 (male)	P{w[+mC]=UAS-Rho1.N19}2.1- **BL7328**
Fig. S3A,B	P{w[+mC]=sqh-Pak1.RBD-GFP}21/+;<br>nos-tdTomato-Anillin-RBD-P2A-tdKatushka2-CAAX/+	P{w[+mC]=sqh-Pak1.RBD-GFP}21- **BL56549**
Fig. S3C,D	P{sqh-WASp.RBD-GFP}378a P{sqh-WASp.RBD-GFP}378b/<br>nos-tdTomato-Anillin-RBD-P2A-tdKatushka2-CAAX	P{sqh-WASp.RBD-GFP}378a,<br>P{sqh-WASp.RBD-GFP}378b - **BL56746**
Fig. S4A	nos-Lifeact-tdTomato-P2A-tdKatushka2-CAAX;<br>P{sqh-GFP.RLC}3	
Fig. S4B	tre1$^{ep\Delta5}$; nos-Lifeact-tdTomato-P2A-tdKatushka2-CAAX; P{sqh-GFP.RLC}3	
Fig. S4C,D	y1 w* cv1 sqhAX3; P{w[+mC]=sqh-GFP.RLC}2; nos-Lifeact-tdTomato-P2A-tdKatushka2-CAAX	
Fig. S5A	MatTub-gal4/UASp-mClover2-Rho1-G14V-*nos* 3'UTR	UASp-mClover2-Rho1-G14V-*nos* 3'UTR- **This study**
Fig. S5B	MatTub-gal4/UASp-mClover2-Rho1-G14V-*gcl* 3'UTR	UASp-mClover2-Rho1-G14V-*gcl* 3'UTR- **This study**
Fig. S5C,D	MatTub-gal4/UASp-mClover2-Rho1-G14V-*pgc* 3'UTR	UASp-mClover2-Rho1-G14V-*pgc* 3'UTR- **This study**
Fig. S5E	MatTub-gal4/UASp-mClover2-Rho1-G14V-*nos* TCE-*pgc* 3'UTR	UASp-mClover2-Rho1-G14V-*nos* TCE-*pgc* 3'UTR- **This study**
Fig. S6	WT- Ecad::3xGFP<br>tre1$^{-/-}$- tre1$^{ep\Delta5}$; Ecad::3xGFP	
	**References**	
	1. Tran, V., Lim, C., Xie, J. & Chen, X. Asymmetric division of Drosophila male germline stem cell shows asymmetric histone distribution. Science (New York, N.Y.) 338, 679-682 (2012).	
	2. Kong, D. et al. In vivo optochemical control of cell contractility at single cell resolution. *EMBO Reports,* EMBR1310	
	3. Pinheiro, D. *et al.* Transmission of cytokinesis forces via E-cadherin dilution and actomyosin flows. *Nature* **545**, 103 (2017).	
	4. Izquierdo, E., Quinkler, T. & De Renzis, S. Guided morphogenesis through optogenetic activation of Rho signalling during early Drosophila embryogenesis. *Nature Communications* **9**, 2366 (2018).	

Supplementary Table 2

**Primer name**	**sequence**	**Purpose**
pwal22_loxp_rev	CATACATTATACGAAGTTATAAGCTGGTACTACTAGT	Create pwalium22 infusion template lacking UAS and K10
pwal22_k10end_fwd	GCGTGCTTTGGAGCTGAG	Create pwalium22 infusion template lacking UAS and K10
pwal22_nheI_nos5utr_fwd	TTCGTATAATGTATGgctagcAAGCTTCGACCGTTTTAACCTCG	Clone *nos* 5'UTR and promoter into pwalium22
nos5utr_ZraI_nos3utr_rev	CAGAGCTGGATTCGCCCTCTgacgtcGGCGAAAATCCGGGTCGAAA	Clone *nos* 5'UTR and promoter into pwalium22
nos3utr_fwd	AGAGGGCGAATCCAGCTC	Clone *nos* 3'UTR into pwalium22
pwal22_ecori_nos3utr_rev	AGCTCCAAAGCACGCgaattcCCTTAAATTGTAACCATTTCTTTATTTGGCACTC	Clone *nos* 3'UTR into pwalium22
Pwalium22_NheI_rev	GCTAGCGGCTGAATATGGGAT	Create pwalium22 infusion template with UAS and lacking K10
pwal22_nheI_gprk2_fwd	tcccatattcagccgctagcATGGAATTAGAGAATATTGTGGCCAATACG	Clone GPRK2 as a spacer into pwalium22 with *nos* TCE-*pgc* 3'UTR
gprk2_zraI_nosTCE_pgc3_fwd	CGACGGTCGAAAGCTGAgacgtcAGAGGGCGAATCCAGCTCTGGAGCAGAGGCTCTGG CAGCTTTTGCAGCGTTTATATAACATGAAATATATATACGCATTCCGATCAAAGCTGGGTTctggacctcccaaaagcc	Clone GPRK2 as a spacer into pwalium22 with *nos* TCE-*pgc* 3'UTR
pwal22_ecori_pgc3UTR_rev	AGCTCCAAAGCACGCgaattcACGATTGCGAATCGAAAATATATTTCTATCTATTTTTTG	Clone the *pgc* 3'UTR into pwalium22
gcl3utr_fwd_224	GCACGTGCTGAGCAGTC	Clone the *gcl* 3'UTR into pwalium22
gcl3utr_ecori_pwal22_rev	CCCTCAGCTCCAAAGCACGCgaattcTTATTAAGTGTAATCTTTAATAAATGTCACTCAAGGTAATGTTACTG	Clone the *gcl* 3'UTR into pwalium22
nos5_zraI_Lifeact_fwd	CGACCCGGATTTTCGCCgacATGGGTGTCGCGGACCTCATCAAGAAGTTCGAGAGTATTTCCAAGGAAGAG agtgcaggtggaagtgctggaggcagtgcaggtggaATGGTGAGCAAGGGCG	Clone lifeact-tdtomato-p2a-tdKatushka2-CAAX into pwalium22 with nos regulatory elements
tdtomato_nostop_p2a_rev	ggggccggggttctcctccacgtcgccggcctgcttcagcagggagaagttggtggcgccCTTGTACAGCTCGTCCATGC	Clone lifeact-tdtomato-p2a-tdKatushka2-CAAX into pwalium22 with nos regulatory elements
p2aoverlap_tdkatushka_fwd	tggaggagaaccccggccccATGGTGGGTGAGGATAGCG	Clone lifeact-tdtomato-p2a-tdKatushka2-CAAX into pwalium22 with nos regulatory elements
tdKatushka2_caax_stop_zraI_nos3utr_	agctggattcgccctctgacTTAcatgatgacacatttcgttttcgactttttcttttttttcttgccatccttgctcatcttttctttGCTGTGCCCCAGTTTGC	Clone lifeact-tdtomato-p2a-tdKatushka2-CAAX into pwalium22 with nos regulatory elements
pw22_nhei_NSlmb-vhhGFP4_fwd	tcccatattcagccgctagcATGATGAAAATGGAGACTGACAAAATAATGGA	Clone degradFP system into pwalium22 with *nos* TCE-*pgc* 3'UTR
NSlmb-vhhGFP4_stop_nosTCE_rev	CAGAGCTGGATTCGCCCTCTgacTTAGCTGGAGACGGTGAC	Clone degradFP system into pwalium22 with *nos* TCE-*pgc* 3'UTR
pw22_nhei_shotgun_fwd	TTCGTATAATGTATGgctagcATGTCCACCAGTGTCCAGC	Clone dE-cadherin-mClover2 into pwalium22 with *nos* TCE-*pgc* 3'UTR
shotgun_linker_rev	tccacctgcactgcctccagcacttccacctgcactGATGCGCCAGCCCTG	Clone dE-cadherin-mClover2 into pwalium22 with *nos* TCE-*pgc* 3'UTR
linker_mclover2_fwd	ctggaggcagtgcaggtggaATGGTGAGCAAGGGCGA	Clone dE-cadherin-mClover2 into pwalium22 with *nos* TCE-*pgc* 3'UTR
mClover2_stop_zraI_NosTCE_rev	CAGAGCTGGATTCGCCCTCTgacttaCTTGTACAGCTCGTCCATGC	Clone dE-cadherin-mClover2 into pwalium22 with *nos* TCE-*pgc* 3'UTR
pw22_nhei_uasp_NRG_fwd	tcccatattcagccgctagcATGTGGCGGCAGTCAA	Clone Neuroglian-mClover2 into pwalium22 with *nos* TCE-*pgc* 3'UTR
NRG_linker_rev	tccacctgcactgcctccagcacttccacctgcactAAGTCCTTTGCGTCCATATTGG	Clone Neuroglian-mClover2 into pwalium22 with *nos* TCE-*pgc* 3'UTR

Rho1G14V_fwd_137	GTCGGCGACGtcGCCTGCGGTA	site directed mutagenesis for creating Rho1 constitutively active
Rho1G14V_rev_138	AATTACCAATTTCTTGCGAATCGTC	site directed mutagenesis for creating Rho1 constitutively active
Rho1T19N_fwd_139	TGCGGTAAAAacTGCCTTCTGATTGTCTTCAGCAAAG	site directed mutagenesis for creating Rho1 dominant negative
Rho1T19N_rev_140	GGCACCGTCGCCGACAAT	site directed mutagenesis for creating Rho1 dominant negative
pwal22_nheI_mclover2_fwd	tcccatattcagccgctagcGCCACCATGGTGAGCAAG	Clone mClover2-Rho1 constructs
mClover2_nostop_linker_rev	tccacctgcactgcctccagcactCTTGTACAGCTCGTCCATGC	Clone mClover2-Rho1 constructs
linker_rho1_fwd	ctggaggcagtgcaggtggaATGACGACGATTCGCAAGAAATTG	Clone mClover2-Rho1 constructs
rho1g14v_stop_nos3utr_rev	cagagctggattcgccctctTTAGAGCAAAAGGCATCTGGTCTTC	Clone mClover2-Rho1 G14V with *nos* 3'UTR
rho1g14v_stop_pgc3utr_rev	ttggcttttgggaggtccagTTAGAGCAAAAGGCATCTGGTCTTC	Clone mClover2-Rho1 G14V with *pgc* 3'UTR
rho1g14v_stop_gcl3utr_rev	gtggactgctcagcacgtgcTTAGAGCAAAAGGCATCTGGTCTTC	Clone mClover2-Rho1 G14V with *gcl* 3'UTR
pac5_fwd_infus	AGACATGATAAGATACATTGATGAGTTTG	Create actin promoter infusion template
pac5_rev_infus	CCGATCCGGGGTCTCT	Create actin promoter infusion template
pAc_mclover2_fwd	ATCCAGAGACCCCGGATCGGGCCACCATGGTGAGCAA	Clone mClover2-Rho1 constructs into actin promoter vector
rho1_stop_sv40pa_rev	CAATGTATCTTATCATGTCTttaGAGCAAAAGGCATCTGGTCTTC	Clone mClover2-Rho1 constructs into actin promoter vector
pw22_atub67c_prom_fwd	TTCGTATAATGTATGgctagcTTGACAAATGAAGCTGTTACCTGTATTAGT	Clone MatTub-Gal4 into pwalium22
atub67c_prom_rev	GCCAATCTGGATGGAGACTACTT	Clone MatTub-Gal4 into pwalium22
atub67c_Gal4_fwd	tagtctccatccagattggcAAGCTGCTGAGTAGTATTGAACAA	Clone MatTub-Gal4 into pwalium22
Gal4_rev	CTACTCCTTCTTTGGGTTCGGT	Clone MatTub-Gal4 into pwalium22
gal4_atub84b_pw22 (Gblock)	ACCCAAAGAAGGAGTAGgacGCGTCACGCCACTTCAACGCTCGATGGGAGCGTCATTGGTGGGCGGGGTAACCGTCGAAATCAGTGTTTACGCTTC CAATCGCAACAAAAAATTCACTGCAACACTGAAAAGCATACGAAAACGATGAAGATTGTACGAGAAACCATAAAGTATTTTATCCACAAAGACACGTATAGCAGAAAAGCCAAGTTAACTCGGCGATAAGTTGTGTACACAAGAATAAAATCGGCCAGATTCAGTGTTGTCAGAAATAAGgtcgaattcGCGTGCTTTGG	Clone MatTub-Gal4 into pwalium22
