# Supplementary material for: Collectively stabilizing and orienting posterior migratory forces disperses cell clusters in vivo
Source: Nat Commun. 2020 Sep 8;11:4477. doi: 10.1038/s41467-020-18185-2 (PMC7479147; doi:10.1038/s41467-020-18185-2)
Supplement: Supplementary file 3 — Description of Additional Supplementary Files [file 41467_2020_18185_MOESM3_ESM.pdf]

Description of Additional Supplementary Files

File Name: **Supplementary Movie 1**
Description: Dynamics of representative WT (left) and *tre1*$^{-/-}$ (right) PGC clusters visualized by expression of nos-lifeact-tdTomato shown as maximum intensity projections starting at stage 9 of embryogenesis. Endoderm EMT occurs at ~20 minutes.

File Name: **Supplementary Movie 2**
Description: A single Z plane of a pair WT PGCs expressing nos-lifeact-tdTomato outlined in cyan separating from a cluster and transmigrating through the endoderm.

File Name: **Supplementary Movie 3**
Description: A single Z plane of WT PGCs expressing nos-lifeact-tdTomato outlined in cyan separating from a cluster with tails detached (left) or retained (right). Note the increase in lifeact-tdTomato intensity at the tail during separation.

File Name: **Supplementary Movie 4**
Description: A single Z plane of a *tre1*$^{-/-}$ PGC expressing nos-lifeact-tdTomato outlined in cyan remaining within the cluster.

File Name: **Supplementary Movie 5**
Description: Dynamics of representative WT (left) and *tre1*$^{-/-}$ (right) PGC clusters expressing nos-lifeact-tdTomato and H2B-GFP (cyan) shown as maximum intensity projections beginning after endoderm EMT. White boxes mark tracked nuclei and individual tracks are shown. Tracks are time encoded from early (cyan) to late (green).

File Name: **Supplementary Movie 6**
Description: A single Z plane of a WT PGC cluster expressing nos-lifeact-tdtomato (left, grey) and myosin II-GFP (right) imaged prior to endoderm EMT.

File Name: **Supplementary Movie 7**
Description: A single Z plane of a *tre1*$^{-/-}$ PGC cluster expressing nos-lifeact-tdtomato (left, grey) and myosin II-GFP (right) imaged prior to endoderm EMT.

File Name: **Supplementary Movie 8**
Description: A single Z plane of a WT PGC cluster expressing nos-lifeact-tdtomato (left, grey) and myosin II-3xGFP (right) imaged during cluster dispersal.

File Name: **Supplementary Movie 9**
Description: Maximum intensity projections of transplanted WT (left) and *tre1*$^{-/-}$ (right) PGCs expressing myosin II-3xGFP migrating within the endoderm prior to endoderm EMT. Cell trajectories are marked with cyan.

File Name: **Supplementary Movie 10**
Description: Cropped maximum intensity projection from supplementary movie S9 of an individual WT PGC expressing myosin II-3xGFP migrating within the endoderm prior to endoderm EMT. Cell trajectory is marked with cyan.

File Name: **Supplementary Movie 11**
Description: Cropped maximum intensity projection from supplementary movie S9 of an individual *tre1*$^{-/-}$ PGC expressing nos-lifeact-tdtomato and myosin II-3xGFP migrating within the endoderm prior to endoderm EMT. Cell trajectory is marked with cyan.

File Name: **Supplementary Movie 12**
Description: A single Z plane of a representative control PGC cluster expressing nos-lifeact-tdTomato (magenta) and myosin II-GFP (green) imaged during separation and transmigration through the endoderm. The movie begins at the onset of endoderm EMT.

File Name: **Supplementary Movie 13**
Description: A single Z plane of myosin II-GFP depleted PGC cluster expressing nos-lifeact-tdTomato (magenta) and myosin II-GFP (green) imaged at the onset of endoderm EMT.

File Name: **Supplementary Movie 14**
Description: A single Z plane of PGCs migrating in the mesoderm expressing mCherry-RhoGEF2-CRY2 (not shown) and CIBN-pmGFP (grey) subjected to a single 950 nm light pulse (green box). Migration is reversed.

File Name: **Supplementary Movie 15**
Description: A single Z plane of control PGCs migrating in the mesoderm solely expressing CIBN-pmGFP (grey) subjected to a single 950 nm light pulse (green box). Migration is unaffected.

File Name: **Supplementary Movie 16**
Description: A single Z plane of a transplanted WT PGC expressing lifeact-tdTomato (grey) and E-cadherin-3xGFP (green) separating from a host PGC cluster expressing lifeact-tdTomato (grey) during stage 9 of embryogenesis.

File Name: **Supplementary Movie 17**
Description: A single Z plane of a transplanted *tre1*$^{-/-}$ PGC expressing lifeact-tdTomato (grey) and E-cadherin-3xGFP (knock in, green) within a host *tre1*$^{-/-}$ PGC cluster expressing lifeact-tdTomato (grey) during stage 9 of embryogenesis.

File Name: **Supplementary Movie 18**
Description: A single Z plane of a WT PGC cluster expressing lifeact-tdTomato (grey, left panel) and a WT PGC cluster expressing lifeact-tdTomato and overexpressing E-cadherin-mClover2 (middle and right panel) during stage 9 of embryogenesis. Endoderm EMT occurs at ~20 minutes.

File Name: **Supplementary Movie 19**
Description: A single Z plane of a WT PGC cluster expressing lifeact-tdTomato (grey, left panel) and a WT PGC cluster expressing lifeact-tdTomato and overexpressing Neuroglian-mClover2 (middle and right panel) during stage 9 of embryogenesis. Endoderm EMT occurs at ~20 minutes.

File Name: **Supplementary Movie 20**
Description: Individual WT PGCs expressing lifeact-tdTomato (magenta) within a *tre1*$^{-/-}$ PGC cluster expressing Moesin-ABD-GFP (green) during stage 9 of embryogenesis.

File Name: **Supplementary Movie 21**
Description: Group of WT PGCs expressing lifeact-tdTomato (magenta) within a *tre1*$^{-/-}$ PGC cluster expressing Moesin-ABD-GFP (green) during stage 9 of embryogenesis.
