# Supplementary material for: Collectively stabilizing and orienting posterior migratory forces disperses cell clusters in vivo
Source: Nat Commun. 2020 Sep 8;11:4477. doi: 10.1038/s41467-020-18185-2 (PMC7479147; doi:10.1038/s41467-020-18185-2)
Supplement: Supplementary file 25 — Reporting Summary [file 41467_2020_18185_MOESM25_ESM.pdf]

nature research

Corresponding author(s): Ruth Lehmann, Benjamin Lin

Last updated by author(s): Jun 29, 2020

Reporting Summary

Nature Research wishes to improve the reproducibility of the work that we publish. This form provides structure for consistency and transparency in reporting. For further information on Nature Research policies, see Authors & Referees and the Editorial Policy Checklist .

Statistics

For all statistical analyses, confirm that the following items are present in the figure legend, table legend, main text, or Methods section.

n/a	Confirmed	
☐	☒	The exact sample size (*n*) for each experimental group/condition, given as a discrete number and unit of measurement
☐	☒	A statement on whether measurements were taken from distinct samples or whether the same sample was measured repeatedly
☐	☒	The statistical test(s) used AND whether they are one- or two-sided<br>*Only common tests should be described solely by name; describe more complex techniques in the Methods section.*
☒	☐	A description of all covariates tested
☒	☐	A description of any assumptions or corrections, such as tests of normality and adjustment for multiple comparisons
☐	☒	A full description of the statistical parameters including central tendency (e.g. means) or other basic estimates (e.g. regression coefficient) AND variation (e.g. standard deviation) or associated estimates of uncertainty (e.g. confidence intervals)
☐	☒	For null hypothesis testing, the test statistic (e.g. *F*, *t*, *r*) with confidence intervals, effect sizes, degrees of freedom and *P* value noted<br>*Give P values as exact values whenever suitable.*
☒	☐	For Bayesian analysis, information on the choice of priors and Markov chain Monte Carlo settings
☒	☐	For hierarchical and complex designs, identification of the appropriate level for tests and full reporting of outcomes
☒	☐	Estimates of effect sizes (e.g. Cohen's *d*, Pearson's *r*), indicating how they were calculated

Our web collection on statistics for biologists contains articles on many of the points above.

Software and code

Policy information about availability of computer code

Data collection	Two photon Imaging data was acquired in PrairieView 4.3.2.24 on a Prairie Ultima (Bruker technologies) and confocal imaging data was acquired in Zen 2.3 (blue edition) on a Zeiss LSM 800.
Data analysis	Imaging data was analyzed in Fiji (ImageJ 1.52p) , Matlab R2016a (Mathworks), and Imaris 8.0.2 (Bitplane). The CANDLE package for Matlab (available at- http://www.bic.mni.mcgill.ca/ServicesSoftwareAdvancedImageProcessingTools/CANDLE/) was utilized for denoising for figure presentation. Data was plotted and statistical comparisons were carried out in GraphPad Prism 8. Custom written scripts were utilized in Matlab and are available at- https://github.com/linb06/PGC-dispersal

For manuscripts utilizing custom algorithms or software that are central to the research but not yet described in published literature, software must be made available to editors/reviewers. We strongly encourage code deposition in a community repository (e.g. GitHub). See the Nature Research guidelines for submitting code & software for further information.

Data

Policy information about availability of data

All manuscripts must include a data availability statement. This statement should provide the following information, where applicable:
- Accession codes, unique identifiers, or web links for publicly available datasets
- A list of figures that have associated raw data
- A description of any restrictions on data availability

All data is available from the corresponding authors upon reasonable request. Raw data from Figs. 1-7 and Figs. S2-S4 are provided as a Source Data file.

Field-specific reporting

Please select the one below that is the best fit for your research. If you are not sure, read the appropriate sections before making your selection.

- [x] Life sciences
- [ ] Behavioural & social sciences
- [ ] Ecological, evolutionary & environmental sciences

For a reference copy of the document with all sections, see nature.com/documents/nr-reporting-summary-flat.pdf

Life sciences study design

All studies must disclose on these points even when the disclosure is negative.

Sample size	No predetermined sample sizes were calculated. All experiments were from n >= 4 embryos and were consistent. All experiments and their relevant controls were carried out on the same or subsequent day to avoid age or environmental specific effects.
Data exclusions	In timelapse experiments, data were excluded due to technical limitations, which included embryo movement out of the plane of focus and bleaching of fluorescent signals, both of which made results uninterpretable. Data were excluded after acquisition because these phenomena could not be predicted a priori.
Replication	All experiments were carried out with at least two independent replicates (Relevant genotypes were crossed at least twice and siblings used as controls). Multiple embryos were analyzed for every genotype. The data was consistent across replications.
Randomization	Experiments were not allocated into random groups because relevant genotypes after genetic crossing were selected for experimentation. Control experiments from age matched siblings were carried out in parallel.
Blinding	The investigators were not blinded to group allocation or analysis because the same investigator was responsible for both. Data was analyzed in an unbiased manner through semi-automated quantification in Matlab.

Reporting for specific materials, systems and methods

We require information from authors about some types of materials, experimental systems and methods used in many studies. Here, indicate whether each material, system or method listed is relevant to your study. If you are not sure if a list item applies to your research, read the appropriate section before selecting a response.

Materials & experimental systems

n/a	Involved in the study	
[ ]	[x]	Antibodies
[x]	[ ]	Eukaryotic cell lines
[x]	[ ]	Palaeontology
[ ]	[x]	Animals and other organisms
[x]	[ ]	Human research participants
[x]	[ ]	Clinical data

Methods

n/a	Involved in the study	
[x]	[ ]	ChIP-seq
[x]	[ ]	Flow cytometry
[x]	[ ]	MRI-based neuroimaging

Antibodies

Antibodies used	Primary Antibodies- Rabbit anti-Vasa (Lehmann lab generated), rabbit anti-Rab5 (1:500, Abcam, ab31261), mouse anti-Rab7 (1:100, Developmental Studies Hybridoma Bank), chicken anti-GFP (1:500, Aves, GFP-1010), goat anti-vasa (1:500, Santa Cruz Biotechnologies, sc-26877).<br>Secondary Antibodies- FITC AffiniPure Donkey Anti-Chicken (703-095-155), Alexa Fluor® 647 AffiniPure Donkey Anti-Goat (705-605-003), Alexa Fluor® 647 AffiniPure Donkey Anti-Rabbit (711-605-152), Cy™3 AffiniPure Donkey Anti-Mouse (715-165-150), Cy™3 AffiniPure Donkey Anti-Rabbit (711-165-152)
Validation	Rabbit anti-Vasa- This antibody was developed in our laboratory and was first utilized in- Ephrussi A and Lehmann R. Induction of germ cell formation by oskar. Nature. 1992;358(6385):387-392. It has been widely used and validated in the Drosophila community.<br><br>rabbit anti-Rab5- This commercially available antibody was developed against a Drosophila Rab5 antigen and validated for IHC in- Huang et al. Endocytic pathway is required for Drosophila toll innate immune signaling. PNAS. 2010; 107(18)8322-8327.<br><br>mouse anti-Rab7- This antibody was developed against a Drosophila Rab7 antigen and was validated by the depositing lab in Riedel et al. An antibody toolkit for the study of membrane traffic in Drosophila Melanogaster. Bio. Open. 2016. 5:987 - 982.<br><br>chicken anti-GFP- Commercially available and validation data is provided on the manufacturers website via western blot analysis and immunohistochemistry from transgenic mice expressing GFP.

goat anti-vasa- This commercially available antibody was developed against a Drosophila Vasa antigen. Validated for use in the Drosophila embryo in- Gavis et al. Dispensability of Nanos mRNA localization for abdominal patterning but not for germ cell development. Mech. Dev. 2008;125(1-2):81-90.

Animals and other organisms

Policy information about studies involving animals; ARRIVE guidelines recommended for reporting animal research

Laboratory animals	Drosophila Melanogaster (3-11 days old), the w1118 strain was used as the wildtype.
Wild animals	This study did not involve wild animals.
Field-collected samples	This study did not involve samples collected from the field.
Ethics oversight	No ethical approval or guidance was required in this study.

Note that full information on the approval of the study protocol must also be provided in the manuscript.
